# Implementation of Quiet Areas in Sweden

**DOI:** 10.3390/ijerph16010134

**Published:** 2019-01-07

**Authors:** Gunnar Cerwén, Frans Mossberg

**Affiliations:** 1 Department of Landscape Architecture, Planning and Management, Swedish University of Agricultural Sciences, 230 53 Alnarp, Sweden; 2 Sound Environment Center, Lund University, 222 10 Lund, Sweden; frans.mossberg@kultur.lu.se

**Keywords:** quiet areas, environmental noise, noise abatement, soundscape design, landscape planning, urban planning, general plan, sustainability

## Abstract

The notion of quiet areas has received increasing attention within the EU in recent years. The EU Environmental Noise Directive (END) of 2002 stipulates that member states should map existing quiet areas and formulate strategies to keep these quiet. Quiet areas could play an important role in balancing densified urban development by ensuring access to relative quietness and associated health benefits. This paper reports on a recent study investigating how the notion of quiet areas has been implemented in Sweden. The study, initiated by the Sound Environment Center in 2017, was carried out in two phases. In phase one, an overview of the current situation was obtained by scrutinizing regional and municipal mapping initiatives, aided by a short digital questionnaire sent out to all 290 municipalities in Sweden. This provided a general understanding and highlighted initiatives for further study in phase two. The results revealed that 41% (*n* = 118) of Sweden’s municipalities include quiet areas in their general plans, but that significantly fewer of these have sophisticated strategies for implementation (*n* = 16; 6%). Moreover, the interest in quiet areas in municipalities does not seem to be directly related to the END, but is instead inspired by previous regional initiatives in Sweden. The study highlights a number of considerations and examples of how quiet areas are approached in Sweden today. In general, Sweden has come a long way in terms of identifying and mapping quiet areas, but more progress is needed in developing strategies to protect, maintain, and publicize quiet areas.

## 1. Introduction

In the EU’s Environmental Noise Directive (END) of 2002 (2002/49/EC) [1], member states are asked to make an inventory of existing quiet areas and devise strategies for their protection. The whole concept of quiet areas could be regarded as a way to turn the question of sound environment around, to focus on potential positive qualities of the sound environment and not only on noise and disturbances. The concept connects to tendencies in cultural and general environmental movements of the time with roots back in the soundscape movement [2] and acknowledges the fact that sound may have positive health effects [3].

Sweden is known as one of the countries in Europe working more actively with quiet areas, including several initiatives on regional and municipal level. This could be related to Sweden’s early implementation of the soundscape perspective in research on environmental noise, not least through the Mistra project “Soundscape for better health” [4], carried out between 2000 and 2007, in which the notion of “quiet façade” was introduced.

Recent exposure data from the European Environment Agency (EEA) demonstrate that more than 100 million European citizens are negatively affected by high noise levels, impacting human health [5]. The World Health Organization estimates that one million healthy life-years in Western Europe are lost annually as a result of exposure to traffic noise [6]. Noise has been shown to have severe negative effects on health, including hearing damage, sleep disturbance, hypertension, and cardiovascular disease [7,8].

The situation in Sweden concerning exposure to traffic noise, and to airborne particles, in the immediate vicinity of permanent dwellings has become increasingly problematic since the introduction of more relaxed noise regulations by the Swedish government in 2015 [9], in response to political pressure [10]. In sharp contrast to the scientific advice from the research community on health and the environment, more noise is now permitted close to people’s dwellings than was the case before the updates to the legislation. This proved to be politically acceptable because of an urgent need for housing, but also because of building industry and business interests. The higher threshold levels now in place mean that, in principle, unlimited noise is permitted on the noisiest side of dwellings in noise-exposed urban areas [9,11]. 

The recently published Environmental Noise Guidelines for the European Region [7] define the aim of these guiding principles as reducing noise while conserving quiet areas. Based on previous research [12,13,14], the report states that “people appreciate quiet areas as beneficial for their health and well-being, especially in urban areas” [7]. A growing number of studies indicate that exposure to natural sounds may have positive health effects by reducing stress [3]. Thus quiet areas not only protect against noise, but also reveal positive sounds that would otherwise be masked by noise. 

Urban areas around the world are currently facing new challenges, as there is increasing demand for densification related to sustainability. In order to safeguard the quality of parks and other recreational spaces in the future, quiet areas are likely to become even more important, not only in cities, but also in the vicinity of cities and in open country. 

There are good reasons to build on the notion of quiet areas, as postulated in the END [1]. The present study investigated how the concept has been implemented in regional and municipal contexts in Sweden. The results may be useful as reference for further implementation of the concept in Sweden and other countries, as well as informing future strategies and guidelines on EU level.

## 2. Materials and Methods 

The study comprised two phases: a quantitative phase providing an overview of the situation in Sweden and a qualitative phase in which chosen examples were studied further.

In phase one, an overview was made and background information on quiet areas in Sweden was obtained by studying government documents and reports dealing with quiet areas. The internet was searched extensively and key individuals were contacted. The outcomes are described in Section 3 of this paper. 

Based on the findings, a subdivision was made into regional and municipal initiatives when selecting examples for further studies. The regional initiatives were studied manually, by going through reports and other documents describing these. A total of 10 initiatives were identified and are described in Section 4. In order to study municipal initiatives, an email was sent out to the registrar of all 290 municipal authorities in Sweden. The email included a short description of the project and a digital questionnaire containing three introductory questions on how the municipality works with quiet areas, which the registrar was asked to redirect to the appropriate department. These questions were intended to provide an overview of the extent to which the concept of quiet areas appears in the municipalities’ general plans (Question 2) and to identify initiatives for further study (Questions 1 and 2). Question 3 was included to obtain contact details. For Questions 1 and 2, a set of pre-given options was provided, plus a box for comments:

1. Is there any area in your municipality that has been designated a quiet area, a noise-free area or anything corresponding to this?
  1a) Yes, in an urban setting. 1b) Yes, in a rural setting. 1c) No, not at all. 1d) Other (comment box).
2. Is “quiet areas” included as a question to be dealt with in your municipality’s general plan?
  2a) Yes, elaborated. 2b) Yes, briefly. 2c) No, our municipality does not have a need for quiet areas. 2d) No, but we have discussed implementation in our future plan. 2e) No, and it has not been up for discussion. 2f) Other (comment box).
3. Is there anyone at the municipality we can contact for follow-up questions? (please supply email address/es).

Within one month, during which two reminders were sent out, 208 of the municipalities had answered (response rate 71.7%).

In order to provide a full overview for question 2, the remaining 82 municipalities were investigated manually by means of a digital search in their respective general plans. The search words used were “quiet” (Swedish: tyst) and “noise-free” (bullerfri). In this way, it was determined whether each of these municipalities deals with quiet areas or not. Moreover, if it was found that the municipality mentioned quiet areas, it was determined whether this mention was brief or comprehensive (corresponding to the alternatives in Question 2). Any other trends or noteworthy issues were entered in an Excel spreadsheet. 

A total of 47 municipalities were identified in phase one as interesting for further study. This selection was based on answers given to the questions and information obtained in the manual search of general plans. Moreover, some initiatives were added to this group based on searches on the internet and tips from the public (the project generated media attention that encouraged some people to make contact).

In phase two of the study, the 47 chosen initiatives were investigated further to obtain more information about how quiet areas are treated in Sweden. Furthermore, efforts were made to identify municipalities that had taken action and to explain why others had not taken any action at all. We also sought to identify and discuss general tendencies, challenges, and future possibilities.

Data were collected in phase two through different methods. Initially, a more detailed questionnaire was sent out to municipalities selected for further study. This questionnaire was sent out digitally to email addresses obtained in response to Question 3 in the first digital questionnaire. The detailed questionnaire consisted of 23 questions that dealt with how the municipality had gained inspiration for its work on quiet areas, if there were any measures to protect the quiet areas, and if these areas were used in marketing and tourism, as well as general questions about the level of knowledge and potential for improvement. After one month and one reminder, 25 out of 47 questionnaires were returned (53.2% response rate). Further correspondence followed in some cases.

Results from both research phases are reported below, starting with an overview of how the notion of quiet areas has developed in Sweden and the EU, and important initiatives and definitions (Section 3). We then go on to describe how the concept is used in Sweden, on regional level (Section 4) and municipal level (Section 5). This is followed by a discussion of general trends and challenges identified (Section 6) and conclusions (Section 7). 

## 3. Quiet Areas: Background and Definitions

In Sweden, the notion of quiet areas can be traced to the late 1990s, when the Swedish Road Administration initiated a pilot project in which two municipalities in Jönköping County were mapped for relative quietness [15]. Within a few years, similar mapping initiatives had been undertaken in two other counties [16,17] and in major urban regions [18,19]. Several of these initiatives were connected to a collaborative project officially launched in 2002 with the purpose of developing a method for inventory of quiet areas [20]. The collaboration involved influential stakeholders, such as the Road Administration, the Rail Administration, the National Board of Housing, the Civil Aviation Administration, the Environmental Protection Agency, and two major county councils. The project finished in 2005 [21], and the outcomes were made accessible in an influential report entitled “A Good Sound Environment—More than Merely Absence of Noise” published by the Swedish Environmental Protection Agency [22].

The method used in the collaborative project was based on the assumption that disturbances vary depending on context, and it considers areas divided into five different noise classes (A–E). Noise class A has the highest demands; it corresponds to areas completely free from community noise, such as remote areas in the mountains, forests, and national parks. The benchmark for noise class A is set at 25 dBA (A-weighted instantaneous sound pressure level), whereas the class with the next-highest demand, noise-class B, has a benchmark of 35 dBA. Noise classes C and D are intended for forests and recreation areas in proximity to urban developments and are both based on a benchmark of 45 dBA (instantaneous), but what is called the “exceeding time” differs. The lowest demands are found in noise class E, where the benchmark is set to 45–50 dBA (equivalent), or 10–20 dBA lower than the surrounding sound pressure level. Noise class E is intended to be applied in urban areas such as parks.

In the EU, the development of quiet areas can be traced back to 1996 and the document “Green Paper on Future Noise Policy” [23], where it is mentioned how noise mapping could be used to identify quiet areas. The thoughts raised in this green paper are enforced in the END [1]. However, the instructions in the END are relatively vague, which has resulted in different interpretations and implementations.

In 2014, “The Good Practice Guide on Quiet Areas” [24] followed up on the END by mapping how the question had been dealt with, through examining existing examples and initiatives. The report provided a good overview of existing initiatives in Europe. No specific recommendations were given, but it was suggested that the review could be used as inspiration and reference was made to “competent authorities” for further guidance.

In 2016, the report “Quiet Areas in Europe: The Environment Unaffected by Noise Pollution” [25] introduced a method called Quietness Suitability Index (QSI) for identifying quiet areas in rural contexts. The QSI approach makes use of existing noise mapping data combined with land use data to identify areas that are potentially quiet. No applications of the method have been made yet in Sweden.

The END [1] distinguishes between two different kinds of quiet areas, “open country” and “agglomerations”, which are defined as follows:

“A quiet area in open country’ shall mean an area, delimited by the competent authority, that is undisturbed by noise from traffic, industry or recreational activities.”

“A quiet area in an agglomeration’ shall mean an area, delimited by the competent authority, for instance which is not exposed to a value of Lden or of another appropriate noise indicator greater than a certain value set by the Member State, from any noise source.”

In both definitions, the END refers to the “competent authority” of the member state. In Sweden, the competent authority can be said to correspond to the collaboration group mentioned above and the noise classes it proposed [22]. Thus in Sweden, the END definition for rural settings can be said to correspond to noise classes A and B, while the END definition for urban settings corresponds to noise class E. Noise classes C and D, are intermediate, suggesting that there is scope for a third type of quiet area, i.e., those in ‘proximity to urban areas’.

The collaboration group’s noise classes have been influential for assessing and mapping quiet areas in Sweden. Many of the municipalities in Sweden refer to the benchmarks suggested in the method. However, there are other values that do not relate to this framework. For instance, two benchmarks, 30 dBA and 40dBA, which are still used relatively frequently, derive from early regional initiatives in Sweden undertaken by the Road Administration [16,17] and an earlier version of the collaboration group’s method [20].

Moreover, it is common for the benchmarks to be adjusted, depending on the context in which the noise classes are applied and the data available. In most of the initiatives studied here, only one or two of the classes were used. Non-acoustic factors were influential when quiet areas were defined, including accessibility, natural qualities, and cultural qualities.

More recently, two new methods have been proposed in Sweden [26,27], as well as the QSI on EU level [25], but it is too soon to assess how they are being applied in Sweden.

## 4. Regional Initiatives in Sweden

**Figure 1:**
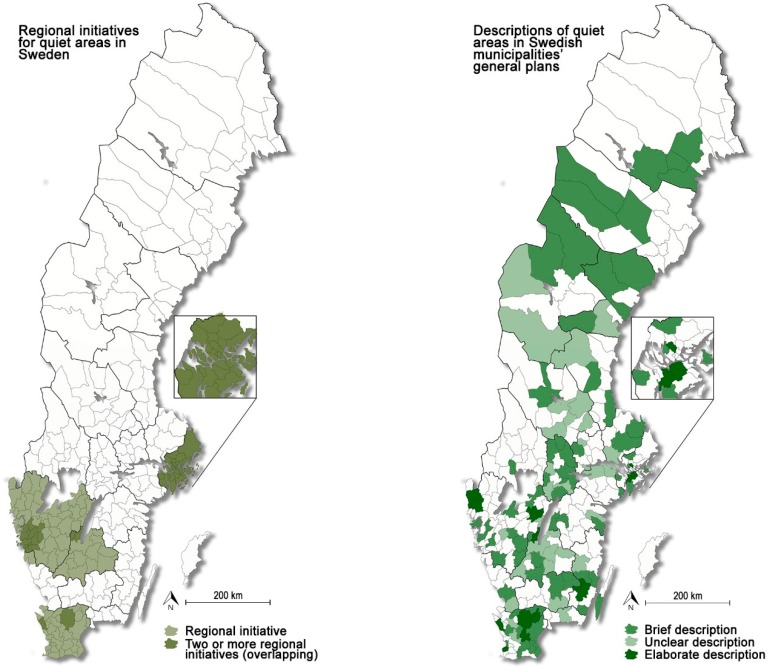
(**a**) Location of regional initiatives for quiet areas in Sweden. Overlapping initiatives are marked with darker color. (**b**) Location of municipalities that describe quiet areas in their general plans, with these descriptions divided into three categories: brief, unclear, and elaborate.

On regional level, a total of 10 initiatives, together covering 121 municipalities, were recorded (see Figure 1a). As can be seen from the map, some of these regional initiatives overlap each other. These areas, indicated with darker color on the map, correspond to some of the most densely populated areas in Sweden, which could be a result of more extensive noise problems in these areas. Moreover, it is interesting to note that, of the 15 municipalities found to have carried out more far-reaching work on quiet areas (see Section 5.1 and Figure 1b), all except one had been preceded by a regional initiative. This suggests that regional initiatives are an important catalyst for implementation of quiet areas.

A short summary of the regional initiatives is presented below, following the chronological order of introduction within the respective region (see Table 1 for an overview).

The first regional initiative was that in Jönköping County by the Swedish Road Administration, in 1998 [15]. Starting from a 30 dBA level equivalent for the noise sources mapped (road, industry, rail, and recreational activities), four quiet areas were identified. Seventeen years later, Jönköping County Council conducted mapping for the whole county on the methodological basis of GIS data [26]. The method was presented as potentially useful for other areas in Sweden as a cost-effective method for modeling larger areas, including vegetation data. By including vegetation data, it was found that it was possible to increase the number of quiet areas. The mapping took account not only of human wellbeing, but also of wildlife. Five categories were introduced, described with words rather than decibel levels. The mapping revealed that 22% of the area of Jönköping County fell within the strictest category, i.e., free from noise.

Västra Götaland County in Western Sweden mapped its quiet areas in 2001 [17]. A benchmark of 30 dBA (equivalent, year) was used and, despite this relatively strict value, it was found that more than half of the area in the county qualified. In that case, the mapping of noise data was coordinated with land use, and quiet areas with experiential potential for nature and recreation were illustrated in maps in a final report. In another initiative in the same county, the municipalities around the Gothenburg region conducted mapping that was published in 2014 [28]. This mapping was based on noise emanating from outside the agglomeration, i.e., roads, rail, air traffic, wind power, industry, and noisy recreational activities. It concerned noise class C (45 dBA instantaneous), as proposed by the collaboration project [22] described in Section 3, but the demands set were higher for some noise sources (large industry, harbor activity, wind power, quarries, dumps, and crushing plants) and equivalent levels were combined with maximum levels as indicators. In all, nine major quiet areas were identified in the region and presented on an illustrated map.

In Scania County, at the southern tip of Sweden, broad mapping was conducted in 2003 based on two benchmarks, 30 dBA and 40 dBA (equivalent levels) [16]. The outcomes were correlated with areas judged to have natural, cultural, and recreational qualities, and these were illustrated in maps. The mapping showed that noise-free areas were rare in Scania County, especially in western parts. Based on this finding, the final report suggested that quiet areas should be valued more highly, particularly if they lie in proximity to urban areas, have connections to other quiet areas, or have high accessibility. For instance, it was found that areas below 30 dBA were generally far away from urban agglomerations, whereas access to areas between 30 dBA and 40 dBA was better. The regional mapping in Scania county was followed up in the next year, when the outcomes were applied in two municipalities, one characterized by an urban setting (Helsingborg) and one characterized by a rural setting (Hässleholm) [29]. In the urban setting, the benchmark 40 dBA was used to identify four areas. In the rural setting, the benchmarks 30 dBA and 40 dBA (equivalent levels) were overlapped with other factors to identify three areas. The municipality found that it was possible to co-locate noisy activities and to use quiet areas to promote the region. In both municipalities, topographical variations were identified as an important factor to consider. 

In the Stockholm area, a number of mappings have been conducted on various regional levels since the turn of the millennium [19,27,30,31]. In the first of these carried out in 2000 [19], the benchmark 45 dBA (equivalent) was used to identify quiet areas in relation to green structures. A few years later, another initiative was taken in which five areas were chosen on the basis that they should both be quiet and have other recreational qualities [30]. Data were extracted through a combination of calculations, measurements, and user interviews. The benchmark 45 dBA (instantaneous) was identified as a threshold for experienced quietness, with a certain exceeding time that varied depending on context. This initiative was linked to the collaboration project mentioned in Section 3 [21]. Another initiative taken at county level proposes a new method for mapping of quiet areas in the region [27]. In addition to these initiatives, the region has a shared development plan in which the notion of quiet areas is emphasized, particularly in relation to green structure [31], and Stockholm County Board has presented a map of quiet areas in the region.

In the Stockholm area, a number of mappings have been conducted on various regional levels since the turn of the millennium [19,27,30,31]. In the first of these carried out in 2000 [19], the benchmark 45 dBA (equivalent) was used to identify quiet areas in relation to green structures. A few years later, another initiative was taken in which five areas were chosen on the basis that they should both be quiet and have other recreational qualities [30]. Data were extracted through a combination of calculations, measurements, and user interviews. The benchmark 45 dBA (instantaneous) was identified as a threshold for experienced quietness, with a certain exceeding time that varied depending on context. This initiative was linked to the collaboration project mentioned in Section 3 [21]. Another initiative taken at county level proposes a new method for mapping of quiet areas in the region [27]. In addition to these initiatives, the region has a shared development plan in which the notion of quiet areas is emphasized, particularly in relation to green structure [31], and Stockholm County Board has presented a map of quiet areas in the region.

## 5. Initiatives on the Municipality Level

The following presentation of municipal initiatives is divided into three parts. The first part provides a quantitative overview of how the notion of quiet areas has been dealt with in Swedish municipalities’ general plans. In the second part, experiences from six municipalities that have progressed further in this work are described. The third part deals with municipalities that have not been working with the concept of quiet areas and looks at the different reasons for this.

### 5.1. Quiet Areas in the General Plans

It was found that 118 of Sweden’s 290 municipalities (41%) mention the concept as part of their strategies in their general plans. Based on how extensive the initiatives were, the municipalities were divided into three categories; brief, unclear, and elaborate descriptions (see Figure 1b and Table 2). These categories are further described below. As indicated in Table 2, the majority of these municipalities focus on rural rather than urban settings. Even though several of these rural initiatives are located in proximity to agglomerations, the lack of focus on urban settings suggest that there is scope to elaborate on quiet areas within such contexts in Sweden.

“Brief descriptions” (*n* = 70; 24%) generally contained a short description and a positive attitude to the notion of quiet areas. Reference was sometimes made to a regional initiative and it was sometimes stated that this issue will be dealt with in future work. Brief descriptions sometimes lacked a definition of how to classify quiet areas and, in most cases, no particular areas were set aside or mapped.

The descriptions of quiet areas provided by 32 of the municipalities (11%) were categorized as “unclear”. In these cases, the term ‘quiet areas’ was confused with other established planning concepts, such as “large unaffected areas” (stora opåverkade områden), “nature reserves” (naturreservat), and “radiation-free areas” (strålningsfria områden).

The descriptions of quiet areas provided by 16 municipalities (6%) in their general plans were categorized as “elaborate”. These descriptions generally included definitions of what a quiet area is, as well as maps of designated areas. In some cases, designated areas were protected and descriptions were provided of how they should be maintained. The 16 municipalities with elaborate descriptions were: Botkyrka, Habo, Helsingborg, Huddinge, Hässleholm, Hörby, Malmö, Munkedal, Nybro, Skövde, Sollentuna, Stockholm, Sundbyberg, Tanum, Tibro, and Östra Göinge. As previously mentioned, 15 of these 16 initiatives (94%) were preceded by a regional initiative (see Figure 1b). In contrast, for the other two categories, brief and unclear descriptions, the corresponding values were 43% (*n* = 30) and 31% (*n* = 10) respectively (Table 3).

This suggests that the regional initiatives were important in inspiring initiatives on municipal level. This was confirmed to some extent by responses to the more detailed questionnaire, where municipalities that had progressed in their work were asked to explain their sources of inspiration. The responses revealed that inspiration from regional mappings accounted for 28% (*n* = 7), while inspiration from neighboring municipalities accounted for 12% (*n* = 3). Local motiving forces emerged as the most important source of inspiration, for 60% (*n* = 15) of the initiatives. Interestingly, only 4% of responding municipalities (*n* = 1) said that the END had been an important factor, suggesting that there is scope for further promotion and/or enforcement of the directive.

### 5.2. Examples of Municipal Initiatives

Initiatives on municipal level were selected for further study with the ambition to focus on examples that could be inspiring for future work, including initiatives that applied more unusual ways of tackling the subject.

**Figure 2:**
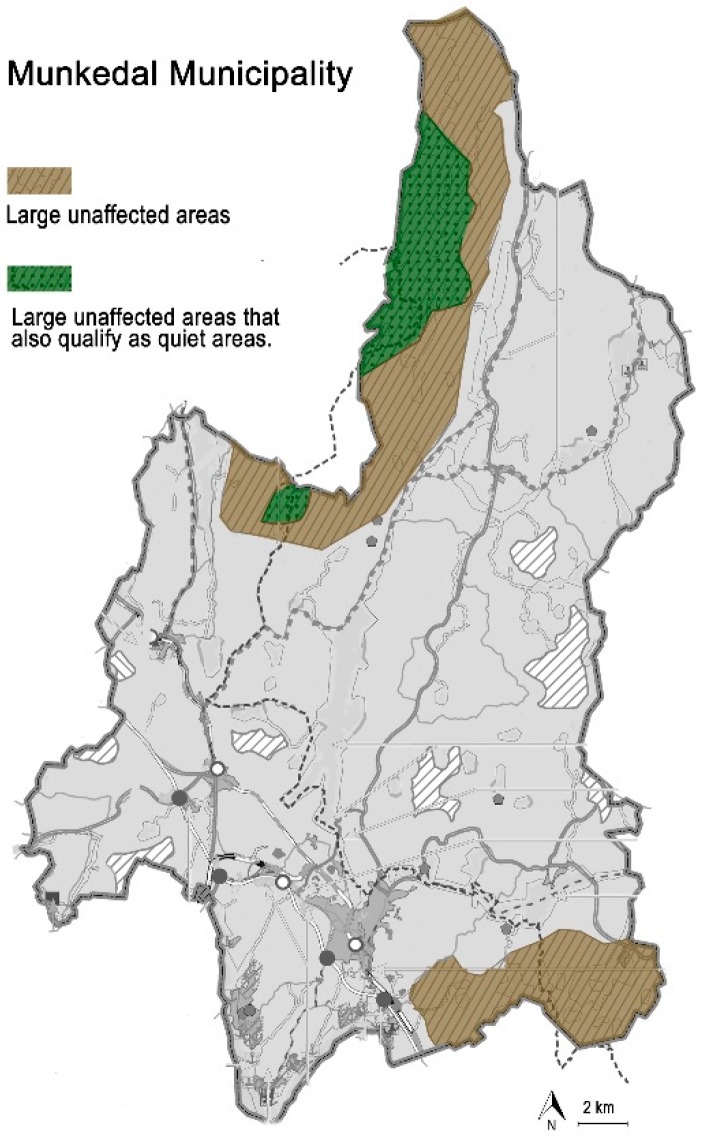
Map of Munkedal municipality, where “quiet areas” overlap with “large unaffected areas”. Adapted and translated from Munkedal’s general plan ÖP14 [32].

Starting in rural locations, the municipality of Munkedal, north of Gothenburg, was covered by regional mapping in 2001 [17]. In the municipality’s general plan, Munkedal ÖP 14 [32], the question is treated on a more detailed level and two quiet areas are designated, both of which border on a wildlife area designated “large unaffected area” (Figure 2). In the plan, the municipality distinguishes between ”unaffected” and ”quiet areas”, but acknowledges overlapping so that certain parts of larger unaffected areas may include quiet areas. This was not the case in some other municipalities, where instead it was assumed that “large unaffected areas” were also quiet areas by definition. The same problem was found in relation to “nature reserves” and other recreational areas.

Nybro, another small municipality in a rural setting, included an addition about quiet areas to its general plan in 2015 [33]. All known sources for noise were mapped. Based on the noise classes proposed by the collaboration group [22], two benchmarks were introduced (35 dBA and 45 dBA, instantaneous). The idea was that by using two different definitions, the various preconditions in the region could be accounted for. After taking account of non-acoustic factors, four areas were proclaimed as free from noise and it was stated that they should remain so. According to the municipality, the designation of quiet areas has already prevented exploitation of wind power in some of these areas. The municipality also says that the areas are maintained through reviewing permits and by supervision, but that more could be done in terms of publicizing them and making them accessible.

**Figure 3:**
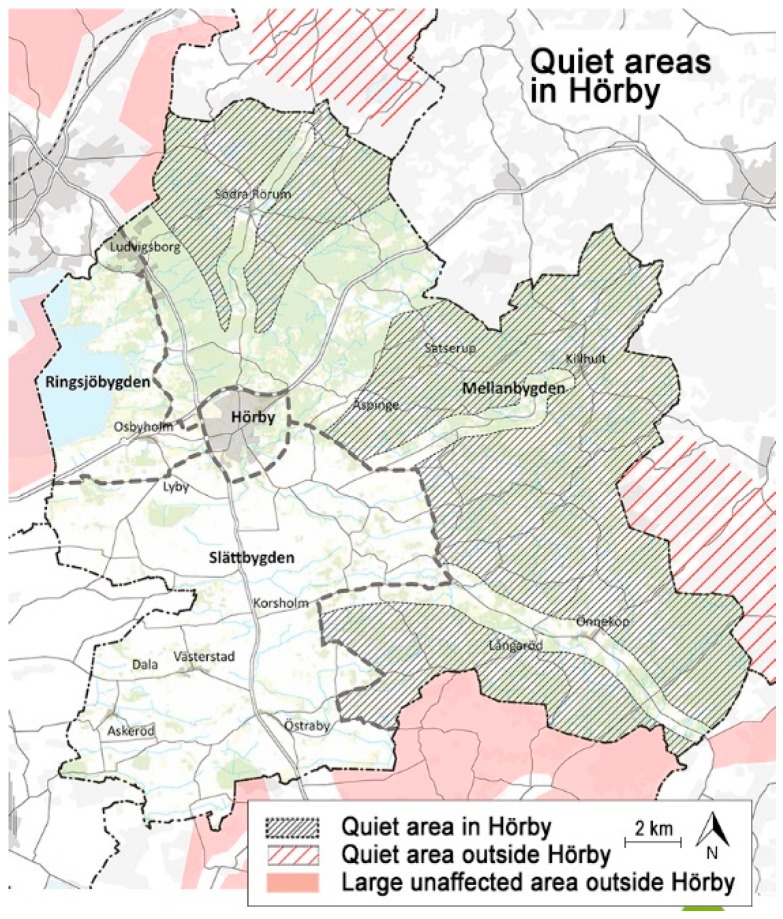
Map of quiet areas in the municipality of Hörby and neighboring municipalities. Adjusted and translated from [34].

In the municipality of Hörby, the initiative to designate quiet areas in the region arose out of activities by the citizens themselves, which were met with positive attitudes by the town council’s planning department. In the current general plan adopted in 2016 [34], parts in the east and north of the municipality are designated free from noise and protected (Figure 3). The definition is based on a previous regional mapping that utilized the benchmark 30 dBA [16]. The general plan includes a detailed account of how the quiet areas are to be protected. For instance, wind farms, shooting ranges, sawmills, and similar activities are to be avoided.

In Stockholm, as previously mentioned, several noise mappings on regional level have been made from the turn of the millennium onwards. In 2013, the concept “Guide to Silence” was introduced in the region, and so far it has been tested in the three municipalities: Sollentuna, Sundbyberg, and Stockholm [35]. The “Guide to Silence” distinguishes itself as the most outgoing of the initiatives found in Sweden. Accessibility is an important aspect and the concept includes publicizing quiet areas on the internet and in illustrated brochures. Signs with maps and symbols have been erected at the sites. According to questionnaire responses from one of the municipalities, Sundbyberg, the concept has been used in marketing the municipality and has received a good response from residents.

**Figure 4:**
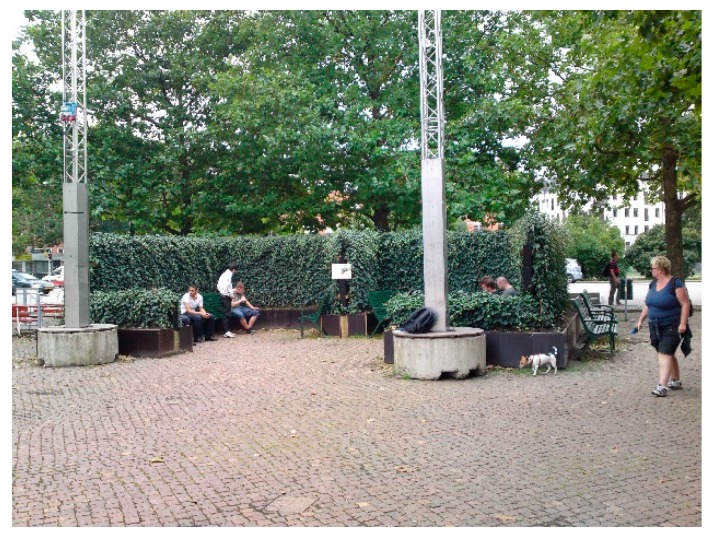
City of Malmö. A small arbor constructed from noise screens covered with ivy. Sounds of nature were added using concealed speakers and visitors’ experiences were evaluated [38]. Photo: Gunnar Cerwén.

The City of Malmö was early to acknowledge quiet areas. Noise measurements of designated areas have been made four times since 1998 [36] and the development can be followed by residents on the City’s website [37]. The results have been quite disappointing since, even though these areas were chosen for their relative quietness, there are extensive problems with noise. An alternative way of working with quiet areas was tested in 2011, when an artificial quiet space was constructed in an urban square (Figure 4). Noise screens covered with ivy were used to form a small and secluded arbor next to a noise-exposed road. It was found that the arbor improved the soundscape compared with outside the arbor, and that this effect was further enhanced when sounds of nature were played inside the space to mask traffic noise [38]. The results illustrated that it is fruitful to combine noise reduction with introduction of masking sounds, thus confirming the fact that it is not only sound pressure levels which are important when discussing quiet areas, but also the quality of the sound.

**Figure 5:**
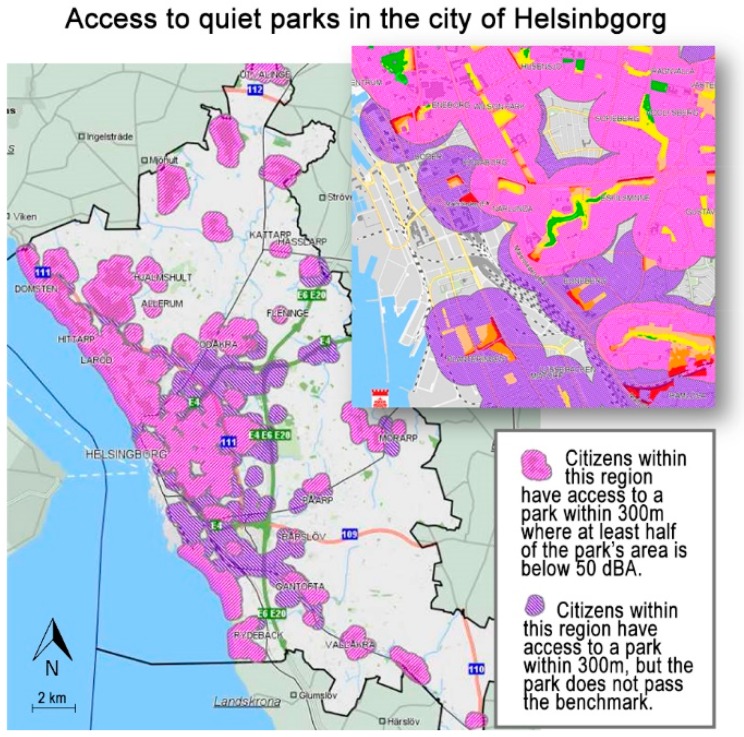
Map showing accessibility of parks and quiet parks in the City of Helsingborg. Adjusted and translated from the City’s action plan against noise [39].

Helsingborg was one of the municipalities that participated in the early initiative undertaken by the Swedish Road Administration in the region [29]. In the municipality’s current general plan, the notion of quiet areas is mentioned briefly, but it is further discussed in the action plan for noise [39]. A benchmark of 40 dBA (equivalent level) is used for rural areas. For parks inside the city, another benchmark is used, incorporating accessibility as a factor. It is thus stated that residents should have a maximum of 300 m to a green area where at least half the area is below 50 dBA. Based on noise data from the City’s parks, accessibility is illustrated in a map (Figure 5). This approach was included here as it illustrates how the notion of quiet areas could be used to ensure variation in a city soundscape. While some urban sounds can be a quality, it is also important to ensure relative quietness [38,40], as this allows residents to choose an environment based on preference, mood, and other factors.

### 5.3. Reasons Why Some Municipalities Are Not Working with Quiet Areas

This section discusses the main reason why some Swedish municipalities do not include quiet areas in their planning. Municipalities that answered no in the questionnaire were given the chance to explain why by ticking boxes. A total of 117 such answers were collected. Among the reasons for lack of activity listed in the questionnaire, “No, our municipality does not have a need for quiet areas” (option 2c) was ticked by 22 municipalities. These municipalities were typically located in remote areas with relatively little human activity and hence good access to quiet areas already. Another 59 municipalities ticked the option “No, and it has not been up for discussion” (option 2e). It was found that the reasons varied, as some municipalities stated that their knowledge was insufficient or that it was an ambiguous term. There were also examples of municipalities, like Salem, which thought that it was useless to work with quiet areas due to the high noise exposure in the region. Another 36 municipalities stated that they had started working on the concept, ticking the option “No, but we have discussed implementation in our future plan” (option 2d), but added that it was not yet implemented in the general plan or that they were about to start work on it soon.

## 6. General Trends, Challenges, and Future Prospects

The final part of the study focused on tendencies and challenges, especially in light of future development, legislation, and implementation.

### 6.1. Confusion Surrounding the Concepts

There is some confusion about the concept of quiet areas as such, as a few different terms are used interchangeably in the Swedish planning community. The terms “quiet areas” (tysta områden) and “noise-free areas” (bullerfria områden) are the most well-established, but there are also other terms, such as “undisturbed areas” (ostörda områden), “carefulness areas” (varsamhetsområden), and “tranquil areas” (lugna områden), that are used more or less synonymously. 

There are several other closely related terms in the planning discourse, including ”large unaffected areas” (stora opåverkade områden), “radiation-free areas” (strålningsfria områden), “consideration areas” (hänsynsområden), ”nature reserves” (naturreservat), “green structure” (grönstruktur) and “areas for recreation” (rekreationsområden). As described previously (see Section 5.1 and Section 5.2), some of these concepts were confused with quiet areas in the survey responses. This was particularly noteworthy for “large unaffected areas”, where it was assumed that these areas were also quiet. For instance, the municipality of Halmstad reported that it had not focused specifically on quiet areas, but that “in the concept of large unaffected areas, the sound environment is naturally included”. However, as the municipality went on to state, in two of the areas identified as “unaffected”, it was evident that only one would qualify as “quiet”. The municipality of Åre, similarly, argues that because a large part of the municipality is protected as a nature reserve, it is probably quiet. 

Assumptions such as these could be problematic and confirm the need for a term like “quiet areas” or “noise-free areas” that focuses specifically on the sonic aspects. Some of the other terms, such as “undisturbed areas” and “tranquil areas”, do not specifically focus on sound and the use of such concepts may increase the confusion further.

It was found that the notion of “quiet areas” can be overlapped with other planning concepts to provide a more nuanced understanding, as illustrated by the municipality Munkedal [32] and its use of the concept (see Section 5.2).

### 6.2. Definitions and Identification of Quiet Areas

Definitions of quiet areas vary widely in Sweden. Most definitions include a reference to a benchmarked sound pressure level, which is derived either from a regional initiative and/or from five different classes of noise exposure developed by a number of influential stakeholders [22]. In Sweden, the noise classes can be said to correspond to what the EU END [1] describes as a “competent authority”. 

The END distinguishes between two main types of quiet areas, i.e., in “open country” and in “agglomerations”. This division was used as a starting point in the present study. It was found that it was significantly more common for Swedish municipalities to work with the concept in open country, suggesting that there is scope to further develop strategies for agglomerations. Such focused initiatives have been undertaken in other parts of Europe [41,42,43,44]. Moreover, the findings suggest that it would be feasible to extend the division to three types of quiet areas (urban, urban proximity, and rural), in order to account for areas that are in close proximity to agglomerations. Such a division would allow for a more nuanced and contextual use of quiet areas, as exemplified in the five noise classes discussed in the study [22].

This study confirms what has already been noted in Europe [24], i.e., that approaches and definitions vary extensively. This is not necessarily a problem given that various contexts set different demands. However, the ambiguities surrounding the concept were reported as being problematic by some municipalities, resulting in a need for clarification. An overview of approaches has previously been published on EU level [24], and the present paper contributes a Swedish perspective. It provides an overview of current methods and approaches in use in Sweden, and this can hopefully provide some guidance for future work. However, an independent evaluation of the many methods that are currently being used would be useful for future research.

The overview on EU level [24] describes four main types of methods to identify quiet areas; noise calculations, noise measurements, evaluations by experts, and evaluations by users (e.g., interviews and surveys). No specific recommendations are presented, except that a combination of these four methods should be used. All four methods were found in Sweden and at least two of the methods were simultaneously put into practice in most cases. Most typically, benchmarks of sound pressure levels were combined with expert evaluations. The experts typically contributed knowledge about aspects such as land use, accessibility, and natural and recreational qualities. Of the four methods, user participation emerged as least commonly applied, although there were examples of its use, e.g., in Upplands Väsby, where interviews were used to identify quiet areas. In future implementations, it is possible that users could be involved in a more cost-effective manner through use of digital tools like smart phone applications (see Section 6.4).

### 6.3. Maintenance and Enforcement

While there are many approaches for identifying and mapping quiet areas, there are fewer examples in Sweden of active maintenance and enforcement of quiet areas. In many cases where mapping of quiet areas had been performed, it has been used mainly as an inventory, possibly with a recommendation to avoid further exploitation in the areas identified, if possible. For instance, the municipality of Växjö reported that it had been 15 years since it performed its inventory and that it had not made any follow-up since then. Sävsjö reported similar experiences, stating that “quiet areas have been mapped, but other than that, it is not something that has come to concrete usage when we make our detailed planning”. 

There were some examples in which quiet areas are explicitly protected in the municipality’s general plans, sometimes with clear instructions on the types of activities and exploitation that should be avoided. One such municipality is Nybro [33], which reported that the designation of quiet areas had been used to limit wind power installations.

The conflict between economic interests, on the one hand, and quietness, on the other, was a recurring theme. In particular, wind power was a question that seemed to upset people, as several such conflicts were noted in the study. The conflicts sometimes extended across regional borders, e.g., the municipality of Grums reported that it had designated an area as quiet, yet experienced disturbance from the neighboring municipality: “One area has been designated (as quiet), but on the other side of the municipal border, that municipality and the county have decided to locate a wind power park (12 mills), because it is in the outskirts and few people are disturbed there.”

Another recurring theme, particularly in Northern Sweden, was regulation of snow scooter traffic. Such enforcements were noted in three northern municipalities: Arjeplog, Dorotea, and Kiruna. The municipality of Umeå reported that it had a ban on motor boats but, apart from this, there were few reports of these kinds of restrictions for private citizens in the study.

A related concept to quiet areas, called “consideration areas”, was developed rather recently in a collaboration between three Swedish counties [45]. Consideration areas are used in designated archipelagos, where visitors are encouraged to be careful and not make noise. Boats are encouraged to drive more slowly and to avoid noisy activities. The concept does not have legal support, but is based on common will and collaboration. The experiences to date are reported to be good [45].

Another related initiative should be mentioned in this context; quiet sections in trains. Originally introduced in the early 2000s, quiet sections are now established and used extensively in public transportation around Sweden. A quiet section is usually a separate compartment, secluded from the rest of the train. Potentially disturbing activities such as conversations and mobile phones are prohibited inside the compartment, which allows travelers to work, read, or relax.

To conclude, even though there are some interesting examples in Sweden in terms of maintenance and enforcement of quiet areas in Sweden, there is much scope for further improvement as most of the examples are from other contexts. Maintenance was noted in the questionnaire by 20% of the municipalities as being one of the greatest future challenges. Some related ideas and concepts from other contexts that are described here could possibly be used to promote future development.

### 6.4. Accessibility and Marketing

This section is related to the Section 6.3 (Maintenance and Enforcement), as both deal with outward activities aimed at the end users. As mentioned, it was found that there was scope for improving accessibility and marketing quiet areas. Mappings of quiet areas described in general plans and other planning documents may be difficult for the end user to access, and it is therefore noteworthy that few municipalities have focused on outward activities. Even municipalities that have come far in their mapping of quiet areas, such as Nybro, reported that there is more to be done in terms of publicizing these areas and making them accessible to the public.

There was a demand for good examples as inspiration in the municipalities’ own work. Relatively few such good examples were identified in the study, with the exception of the “Guide to Silence” in the Stockholm region (see Section 5.2). A related initiative has been developed in the UK and is called “Tranquility Trails” [44].

The internet can be an effective way to reach residents, via channels already established by the municipalities and via marketing on independent websites. No dedicated apps for smartphones were reported to have been developed in Sweden, but an application, Hush City, in the EU allows users to measure sound pressure levels, document favorite places, and inform other users about their favorite quiet areas [41]. It is also possible to plot quiet trails in Google maps.

**Figure 6:**
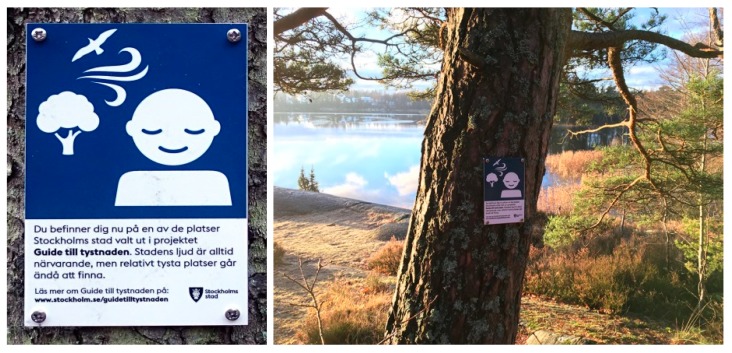
In the “Guide to Silence” concept, quiet areas are marked by signs. This one reads: “You are now in one of the places chosen by the City of Stockholm for the project “Guide to Silence”. The sounds of the city are always present, but relative quietness can be found.” Photo and illustration: Ulf Bohman.

Signs are another way to raise awareness about quiet areas. This is used in the “Guide to Silence” (Figure 6), where each trail’s starting point is marked with a large sign containing a map and a short description of the concept [35]. Moreover, a dedicated symbol is placed at strategic locations. Signs are also used in the related concept of “consideration areas” [45]. 

The spatial distribution of quiet areas is an important factor for accessibility and one that can be controlled in planning. In Sweden, it is generally suggested that urban dwellers should be at most 300 m (about 5 min’ walking distance) from the nearest green area or park [46]. This recommendation has been discussed as a possible benchmark for quiet areas in urban regions [40,47] and has been applied in the City of Helsingborg (see Section 5.2) [39]. Spatial distribution is a factor that could be given more attention, in particular as it lends itself to quantification. However, for rural regions, no corresponding recommendations were encountered. It can be assumed that the quieter an area is, the farther people are willing to travel to get there. Thus, quiet areas in urban regions compensate for the relative noise exposure by proximity, while quiet areas in open country do the opposite. In formulation of future recommendations, it is possible that multifractal modeling could be used as inspiration [48], i.e., that the distribution could be varied depending on type of quiet area and relationships between different types of quiet areas.

Designation of quiet areas is sometimes associated with economic conflicts, as activities like wind power and other industries cannot exist in the proximity of quiet areas. However, there is also the possibility of positive repercussions for the economy if quiet areas are used in marketing. For instance, in the City of Malmö it is clear that the income levels of residents in different neighborhoods correlate with freedom from noise disturbance [49]. Quietness thus appears to be an experiential quality that can be valued economically. There is potential to use quiet areas to create added value by attracting inhabitants and tourists.

**Figure 7:**
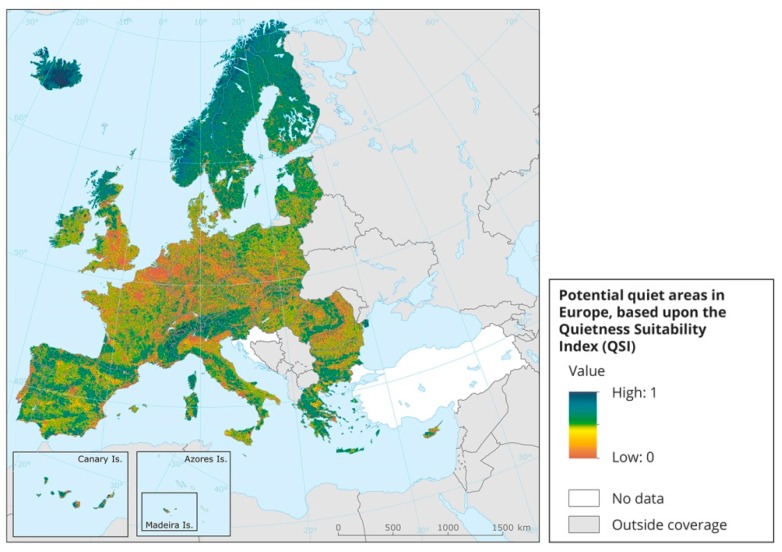
Map of Europe created using the Quietness Suitability Index (QSI), where the potential for quiet areas is based on noise data and land use. Map previously published by the European Environmental Agency [25].

A survey conducted at EU level [25] using the Quietness Suitability Index (QSI) has shown that the Nordic region has relatively good access to quiet environments (see Figure 7). The access to quietness could thus be used as a potential tool in tourism-related marketing. However, only a few examples were found where quietness was marketed in this way, suggesting that there is scope for development in this respect. This could result in economic benefits and also serve as a way to highlight and value quiet areas further, thus providing a means by which to protect them in the future.

## 7. Conclusions 

Although quiet areas are becoming rare in some urban regions in Sweden, it can be concluded that there is growing interest in preserving existing quiet areas. It was found that the concept is well-known in the Swedish planning community, but that few municipalities have been dealing with it in a comprehensive manner. This seems to be due to ambiguities surrounding the concept and lack of good examples. Continuous revision and development of strategies for working with quiet areas is suggested. The study identified a total of 16 municipalities in Sweden that have come further in their work than others and experiences from some of these are described.

The results do not indicate that the EU’s Environmental Noise Directive (END) has had a significant impact on the development of quiet areas on municipal level. A more important factor in Sweden seems to be various regional initiatives undertaken in recent decades. The municipality’s own driving force and inspiration from neighboring municipalities also appear to be important factors.

A number of challenges to future work on quiet areas, in Sweden and at EU level, were identified. In Sweden, many initiatives have focused on mapping and identification of quiet areas, but less has been done regarding maintenance and enforcement. If the principles stated in the recent World Health Organization guidelines on reducing noise while conserving quiet areas are to be followed, more attention definitely needs to be given to such aspects. Some activities are more problematic than others as regards noise emissions, such as snow scooter use in open country, motorsports, and wind farms. In a few cases, activities like these were specifically described in the environmental strategies of some Swedish municipalities as unwanted and undesirable. However, examples of limitations such as these affecting individual activities were fairly rare in the survey data.

There were significant differences in how municipalities work with quiet areas. Such differences are not necessarily problematic per se, as the prerequisites for quiet areas vary depending on context, but it was found that further clarification and guidelines could benefit applicability. In particular, there is a need for an independent evaluation of the methods and definitions available. In the END, a distinction between two types of quiet areas is proposed (agglomerations and open country), but our results indicate that this could be extended to three types: urban areas (agglomerations), areas in urban proximity, and rural areas (open country).

There were very few examples of public communications and marketing of quiet areas by Swedish municipalities, although quiet areas can be beneficial for residents and for tourism, i.e., they are beneficial for health, quality of life, and the local economy. Many parts of Northern Europe, including Sweden, provide rich access to quiet areas and this could play a more prominent role in marketing the country. In addition to economic benefits from tourism, this could also be a way to raise the value and awareness of quiet recreational areas so that they are protected and maintained in the future.

## Figures and Tables

**Table 1:** Overview of regional ‘quiet area’ initiatives in Sweden.

Region:	Year:	Term Used:	Type of Setting:	Benchmark:	Identified Area/s:
Jönköping County [15]	1998	Quiet areas	Rural	30 dBA (equivalent)	4 areas
Jönköping County [26]	2015	Undisturbed areas	Rural/Urban proximity	Undisturbed (approximately 40 dBA)	Illustrated coverage (22% of county area)
Västra Götaland County [17]	2001	Quiet areas	Rural	30 dBA (equivalent)	Illustrated coverage (at least half of county area)
Gothenburg region [28]	2014	Quiet areas	Rural/Urban proximity	45 dBA (max and equivalent), stricter for some events	9 areas
Scania County [16]	2003	Noise free areas	Rural/Urban proximity	30 dBA and 40 dBA (equivalent)	Illustrated coverage in maps
Scania County [29]	2004	Noise free areas	Rural/Urban	40 dBA (equivalent)	7 areas (4 urban and 3 rural)
Stockholm region [19]	2000	Quiet areas	Urban proximity	45 dBA (equivalent, year)	Quiet areas in green wedges
Stockholm region [30]	2005	Quiet areas	Urban proximity	45 dBA (instantaneous)	5 areas studied
Stockholm region [31]	2010	Quiet areas	Urban proximity	45 dBA (equivalent)	Illustrated coverage in maps
Stockholm County [27]	2016	Noise free areas	Rural/Urban/Urban proximity	Four noise classes (20–40 dBA equivalent day/evening)	Proposed methodology for mapping

**Table 2:** Overview of municipal initiatives in Sweden, highlighting the context focused upon in the initiative (urban and/or rural).

Type of Initiative:	Urban Setting:	Rural Setting:	Urban and Rural Setting:	Setting not Known or not Applicable:	Total:
Brief description:	0	33	0	37	70
Unclear description:	0	20	0	12	32
Elaborated description:	1	10	5	0	16
Total:	1 (1%)	63 (53%)	5 (4%)	49 (42%)	118 (100%)

**Table 3:** Number and percentage of municipal initiatives that were preceded by a regional initiative.

Type of Initiative:	Number of Municipalities:	Number (and Proportion) Preceded by a Regional Initiative:
Brief description:	70	30 (43%)
Unclear description:	32	10 (31%)
Elaborated description:	16	15 (94%)
Total:	118	55 (47%)
